# Beliefs and attitudes towards lifestyle change and risks in primary care – a community-based study

**DOI:** 10.1186/s12889-019-7377-x

**Published:** 2019-08-05

**Authors:** Pekka Mäntyselkä, Hannu Kautiainen, Juhani Miettola

**Affiliations:** 1 Institute of Public Health and Clinical Nutrition (General Practice), School of Medicine, University of Eastern Finland, 70211-FIN, P.O.Box 1627, Kuopio, Finland; 2 Primary Health Care Unit, Kuopio University Hospital, Kuopio, Finland; 3 Folkhälsan Research Center, Helsinki, Finland

**Keywords:** Health promotion, Lifestyle, Primary health care, Risk reduction behavior, Attitude to health

## Abstract

**Background:**

Promoting a positive lifestyle change is a challenge for primary health care. The aim of this study was to analyze health and risk-related beliefs and attitudes in relation to lifestyle and lifestyle change in a rural community.

**Methods:**

The study was based on a five-year follow-up data of the Lapinlahti study (*N* = 361). The same structured questionnaire was used at baseline and follow-up with lifestyle items. These were ranked as unhealthy (− 1), neutral (0) or healthy (+ 1). At baseline, participants took a stand on 29 statements related to beliefs and attitudes towards health and health promotion on a 5-point Likert scale. A factor analysis yielded two attitude factors (Factor 1 = underrating risks/resistant to change); (Factor 2 = helplessness/pessimism). The factors were divided into tertiles.

**Results:**

There was a linear positive trend (*P* < 0.001) in baseline lifestyle scores between the tertiles of Factor 1. A positive follow-up change of lifestyle score was found in all tertiles of Factor 1. For Factor 2, the difference between tertiles at baseline was non-significant. There was a significant positive change in all tertiles of Factor 2. Those who were underrating/ resistant but not helpless/pessimistic had the most significant positive lifestyle change. Those who were underrating/resistant and helpless/pessimistic did not improve their lifestyles.

**Conclusions:**

Beliefs and attitudes are related to lifestyle. Subjects with underrating and resistant attitudes with pessimism/helplessness seem to have a low potential for lifestyle change while those with resistant attitudes without pessimism and helplessness may have the most significant potential for lifestyle change. These findings suggest that it is possible to identify different groups of people with different needs and readiness and ability for health behavior change.

## Background

Promoting health and well-being is among the core competencies of primary care [1]. However, health promotion is challenging [2]; alcohol use, tobacco use, high blood pressure, high body mass index (BMI), high cholesterol, high blood glucose, low fruit and vegetable intake, and physical inactivity account for 61% of cardiovascular deaths [3]. The European Network on Prevention and Health Promotion (EUROPREV) [4] study indicated that of the participants with an assessed need for lifestyle change, 10–31% were willing and 13–60% were confident that they could succeed depending on the particular lifestyle issue. The authors concluded that special attention should be paid to men, patients over 50 years of age, and people who rarely go to a general practice.

The scientific knowledge of behavior change is complex. Some types of behaviors, such as smoking, can enhance risks while others, such as exercise, can be seen more as promoting health, and some behaviors may be a part of disease treatment (such as weight control in diabetes). Some may be more related to the social environment (e.g. regular alcohol drinking) or determined by culture (dietary habits) [5]. Furthermore, other background factors such as socioeconomic status are associated with different attitudes to health and risks [6]. Theories of behavior can be classified according to their key determinants contained in the model, e.g., values, attitudes, self-efficacy, habits, emotions or whether they focus on understanding or changing behavior [7]. Some people tend to have a better ability and confidence than others in changing their lifestyles [4, 8]. A persons’ readiness to plan may vary a lot and they may have more or less positive expectations regarding the lifestyle choices [9]. Different people perceive the risks and aspects of health behavior in a different way. According to Health Action Process Approach (HAPA) the change in health behavior is dependent on awareness of risk, outcome expectancy, self-efficacy, intention, and action planning and action control [10].

It has been suggested that in order to be more effective, health promotion should be more personalized and adapted more to individual characteristics and readiness for health behavior change [8]. Because of the complexity in the health behavior of the population, there is an evident need for individual assessment and goal setting, educating and training, and following up on the subjects to whom the lifestyle interventions are targeted [11]. The epidemiological evidence or medical knowledge oriented toward risks and dangers alone may not be efficient in health promotion. Instead, the questions and concerns of respondents and the positive aspects of lifestyle change may provide greater and more sustainable results [12].

The aim of the present study was to analyze how beliefs and attitudes related to health and risk of disease are associated with lifestyle and a change in lifestyle during a 5-year follow-up in the population of a semi-rural community.

## Methods

Lapinlahti is a typical semi-rural municipality in Eastern Finland. The population of the municipality of Lapinlahti was 7500 during the baseline of this study. The baseline Lapinlahti 2005 study involved all 760 adults born in 1939, − 44, − 49, − 54, − 59, − 64, − 69 and − 74 living in municipality of Lapinlahti in Eastern Finland [13, 14]. Of the sample, 594 (78%) responded satisfactorily to a postal questionnaire in 2004. All the respondents were invited to a complete a health survey, which consisted of a structured interview and a health examination conducted by a trained research nurse. At the baseline in 2005, 480 subjects (230 men and 250 women) underwent a complete health survey (baseline study) that consisted of a structured questionnaire and a health check with basic laboratory tests [13]. After the health check at the baseline, all participants were sent a written feedback by the researcher (physician). If necessary, it included advice to e.g. quit smoking, decrease alcohol use and to eat more vegetables, fruits and berries and exercise more. The feedback was formulated by the results of the health examination. No additional individualized feedback or intervention was given.

The present study is based on a five-year follow-up of the baseline cohort [14]. The complete data was available for 361 individuals (males, *N* = 181). The health examination conducted at the baseline (in 2005) and follow-up (in 2010) included measurements of weight and height, blood pressure and waist circumference, and basic laboratory tests. Body mass index (BMI) was calculated as kg/m^2^. Depressive symptoms were assessed using the 21-item Beck Depression Inventory (BDI-21), and a BDI-21 score of 10 was used as the cut-off point for depressive symptoms [15].

At the baseline, all participants filled out a structured questionnaire including 29 statements related to beliefs and attitudes towards health and health promotion on a 5-point Likert scale (1 = totally agree, 2 = agree to some extent, 3 = not applicable, 4 = disagree to some extent and 5 = totally disagree) [16]. Similar statements have been used in the North Karelian project since 1972 and the World Health Organization MONICA (Monitoring trends and determinants in cardiovascular disease) project [17].

At both the baseline and follow-up, the questionnaire included lifestyle items regarding smoking, alcohol use, exercise and nutrition. Based on the national guidelines and health style recommendations, we ranked each of the lifestyle components (smoking, use of alcohol, physical exercise, nutrition) as unhealthy (− 1), neutral (0) or healthy (+ 1). More specifically, we ranked lifestyle as follows: meal beverage (+ 1 = water or non-fat milk, 0 = skimmed milk or sour milk, − 1 = fatty milk or something else), cooking fat (+ 1 = nothing or margarine, dairy spread, 0 = mixture of butter and vegetable oils, − 1 = butter or something else), spreads (+ 1 = nothing or margarine spread, 0 = mixture of butter and vegetable oils, − 1 = butter or something else); use of vegetables (+ 1 = used more than 6 times/week, 0 = used from 1 to 5 times /week, − 1 = never or occasional); berry and fruit intake (+ 1 = used more than 6 times/week, 0 = from 1 to 5 times /week, − 1 = never or occasionally); adding salt to food (+ 1 = never, 0 = usually when food doesn’t taste salty enough, − 1 = often even without tasting); alcohol consumption (male: + 1 = less than 5 doses/week, − 1 = over 5 doses/week or more, female: + 1 = less than 4 doses/week and − 1 = 4 doses/week or more); smoking (+ 1 = never smoking, − 1 = regular or irregular smoking); exercise (+ 1 = daily or more often, 0 = from 1 to 6 times /week,− 1 = less than one time/week). Cooking fat and spreads were combined into a new single variable and the corresponding procedure was conducted with vegetable, berry and fruit (VBF) intake. Thus, we had seven lifestyle items (smoking, alcohol, exercise, food fat, VBF, food fat and salt). These values were summed up and a mean value (ranging from − 1 to + 1) was calculated for each participant. The lifestyle score is described in more detail in the previous article [14].

### Statistical analysis

Statistical significance for the hypotheses of linearity was evaluated by analysis of variance (ANOVA) and Cochran-Armitage tests. When adjusting for confounding factors, analysis of covariance (ANCOVA) models were applied. Collinearity was checked using the variance inflation factor. An exploratory factor analysis with a maximum likelihood method for factoring and orthogonal (varimax) on the polychoric correlation matrix was performed to identify related items of lifestyle [18]. The computed factor scores for the rotated loading matrix divided them into tertiles for further analysis in the shared frailty model. The strategies used to extract the number of factors were the Kaiser criteria, which determines that components with eigenvalues lower than one should be excluded, and the scree test of Cattell criteria. Internal consistency was estimated by calculating Cronbach’s alpha internal consistency with bias-corrected bootstrap 95% confidence intervals. Correlation coefficients were calculated by the Pearson method. The normality of the variables was tested by using the Shapiro-Wilk W test. The Stata 14.1, StataCorp LP (College Station, TX, USA) statistical package was used for the analysis.

## Results

Factor analyses identified 15 statements for two different health belief and attitude factors that explained 89% of the total variance (Table 1). Factor 1 can be defined as underrating risks and negative (resisting) attitude towards health promotion (Factor 1 = “underrating/ resistant”). Factor 2 can be defined as helplessness and pessimism towards health behavior change (Factor 2 = “helplessness/pessimism”). Cronbach’s alpha for the items in Factor 1 was 0.70 (95% CI, 0.65–0.74) and respectively 0.69 (95% CI, 0.61–0.74) in Factor 2. Both factors were divided into tertiles. In Factor 1 (underrating/resistant), tertile I represents the most underrating/resistant subjects and tertile III represents the least underrating/resistant participants. In factor 2 (helplessness/pessimism) tertile I represents the most helpless/pessimistic and tertile III the least helpless/pessimistic participants.

**Table 1:** Factor analysis with varimax loadings of the lifestyle Items

Variable	Factor 1: underrating/resistant	Factor 2: helplessness/pessimism
6, My lifestyle is no one else’s business	0.52	
7, Food with little salt is tasteless	0.48	
10, Smoking is not as dangerous as argued	0.49	
11, Doctors and nurses push too much health advice	0.66	
12, Media pushes too much health advice	0.67	
15, I can’t be bothered to exercise enough to control my weight	0.36	
19, Risk of fatty food are exaggerated	0.57	
25, I find it is difficult for me to choose healthy foods from a grocery store	0.46	
29, Obesity has nothing to do with getting diseases	0.49	
2, My family members don’t support me in my health promotion		0.36
3, I can’t do anything about my excess weight since it is hereditary		0.68
4, I can’t reduce my weight since food is one of my few enjoyments		0.48
20, Doctors cannot give good advice for reducing my weight		0.75
23, I’ve tried my best to lose my weight		0.74
24, Nurses cannot give good advice for reducing my weight		0.35

Coefficients with values < 0.35 not shown

Factors explained 89% of the total variance

The demographic and clinical characteristics according to Factor 1 (underrating/resistant) and Factor 2 (helplessness/pessimism) are presented in Tables 2 and 3. In Factor 1 (Table 2), the proportion of women was the biggest in the least underrating/resistant tertile (tertile III). The same linear trend was found in education indicating that the subjects who were most underrating/resistant had the lowest level of education. An opposite linear trend was found in BMI, glucose, females’ triglycerides, systolic blood pressure, BDI score, cholesterol-lowering medication, and current smoking, indicating the poorest risk factor profile and the unhealthiest lifestyle among the most underrating/resistant participants. The leisure-time physical activity was the highest in tertile III. In Factor 2 (Table 3) the proportion of women decreased towards tertile III, with the proportion of women being the biggest in the most helpless/pessimistic tertile (tertile I). A non-favorable (lower) high density lipoprotein (HDL) cholesterol level and a higher BMI were associated with decreasing helplessness/pessimism. A decreasing trend of depressive symptoms was related to decreasing helplessness/pessimism. The least helpless/pessimistic (tertile III) had the highest level of employment.

**Table 2:** Characteristics of the participants at the baseline according to tertiles of Factor 1: underrating/resistant

	Factor 1: underrating/ resistant Tertiles			*P*-value*
	I (< 70) *N* = 119	II (70–84) *N* = 111	III (> = 85) *N* = 131	
Demographic				
Females, n (%)	44 (37)	47 (42)	89 (68)	< 0.001
Age, years, mean (SD)	52 (10)	49 (10)	51 (10)	0.54
Education: years, mean (SD)	10.1 (2.8)	10.8 (2.9)	11.4 (2.9)	< 0.001
Education: university or university of applied sciences, n (%)	28 (24)	32 (29)	49 (37)	0.017
Living alone, n (%)	14 (12)	10 (9)	15 (12)	0.97
Employed, n (%)	69 (58)	76 (68)	82 (63)	0.48
Clinical				
Body mass index, kg/m^2^, mean (SD)	28.2 (5.4)	29.0 (5.7)	26.7 (4.6)	0.022
Fasting plasma glucose, mmol/l, mean (SD)	5.78 (1.47)	5.58 (0.85)	5.34 (0.75)	0.0041
Serum total cholesterol, mmol/l, mean (SD)	5.23 (1.25)	5.26 (1.08)	5.19 (1.21)	0.78
Serum HDL-cholesterol, mmol/l, mean (SD)				
Male	1.10 (0.38)	1.03 (0.31)	1.13 (0.35)	0.61
Female	1.33 (0.50)	1.40 (0.37)	1.38 (0.39)	0.79
Triglycerides, mmol/l mean (SD)				
Male	1.49 (0.94)	1.57 (0.94)	1.48 (0.89)	0.94
Female	1.35 (0.61)	1.36 (0.97)	1.10 (0.60)	0.012
Blood pressure mm/Hg, mean (SD)				
Systolic	143 (19)	141 (20)	136 (17)	0.002
Diastolic	83 (12)	84 (11)	82 (10)	0.39
Beck Depression Inventory (BDI), mean (SD)	6.9 (6.8)	6.5 (6.7)	4.3 (5.7)	0.0017
BDI ≥10, n (%)	30 (25)	27 (24)	13 (10)	0.002
Current medication				
Hypertension, n (%)	35 (29)	19 (17)	26 (19)	0.076
Cholesterol-lowering, n (%)	22 (18)	14 (13)	12 (9)	0.031
Diabetes mellitus, n (%)	6 (5)	6 (5)	4 (3)	0.44
Current smoking, n (%)	47 (40)	28 (26)	14 (11)	< 0.001
Leisure-time physical activity, n (%)				< 0.001
Low	33 (28)	23 (21)	8 (6)	
Medium	37 (32)	29 (26)	37 (24)	
High	47 (40)	58 (53)	86 (66)	

*P for linearity

**Table 3:** Characteristics of the participants at the baseline according to tertiles of Factor 2: helplessness/pessimism

	Factor 2: helplessness/pessimism Tertiles			*P*-value*
	I (< 60) *N* = 119	II (60–80) *N* = 111	III (> 80) *N* = 131	
Demographic				
Females, n (%)	67 (60)	54 (45)	59 (45)	0.024
Age, years, mean (SD)	50 (10)	51 (10)	50 (9)	0.66
Education: years, mean (SD)	10.8 (3.0)	10.6 (2.9)	10.8 (2.8)	0.90
Education: university or university applied sciences, n (%)	32 (29)	41 (34)	36 (28)	0.81
Living alone, n (%)	12 (11)	14 (12)	13 (10)	0.97
Employed, n (%)	59 (53)	70 (58)	98 (75)	< 0.001
Clinical				
Body mass index, kg/m^2^, mean (SD)	26.3 (6.2)	28.2 (4.9)	29.1 (4.4)	< 0.001
Fasting plasma glucose, mmol/l, mean (SD)	5.48 (1.38)	5.53 (0.76)	5.65 (1.04)	0.28
Serum total cholesterol, mmol/l, mean (SD)	5.11 (1.07)	5.32 (1.26)	5.24 (1.19)	0.42
HDL-cholesterol, mmol/l, mean (SD)				
Male	1.18 (0.38)	1.07 (0.36)	1.04 (0.31)	0.043
Female	1.48 (0.41)	1.36 (0.42)	1.25 (0.38)	< 0.001
Triglycerides, mmol/l mean (SD)				
Male	1.36 (0.84)	1.48 (0.81)	1.64 (1.06)	0.12
Female	1.16 (0.85)	1.28 (0.67)	1.26 (0.61)	0.44
Blood pressure mm/Hg, mean (SD)				
Systolic	137 (21)	141 (18)	141 (17)	0.14
Diastolic	82 (12)	84 (11)	84 (10)	0.13
Beck Depression Inventory (BDI), mean (SD)	7.1 (7.0)	5.8 (6.1)	4.3 (5.9)	< 0.001
BDI ≥10, n (%)	33 (30)	21 (17)	16 (12)	< 0.001
Current medication				
Hypertension, n (%)	25 (23)	24 (20)	31 (24)	0.78
Cholesterol-lowering, n (%)	18 (16)	17 (14)	13 (10)	0.15
Diabetes mellitus, n (%)	4 (4)	7 (6)	5 (4)	0.96
Current smoking, n (%)	30 (28)	30 (25)	29 (22)	0.35
Leisure-time physical activity, n (%)				0.65
Low	22 (20)	19 (16)	23 (18)	
Medium	28 (26)	32 (27)	43 (33)	
High	59 (54)	68 (57)	64 (49)	

*P for linearity

**Fig. 1:**
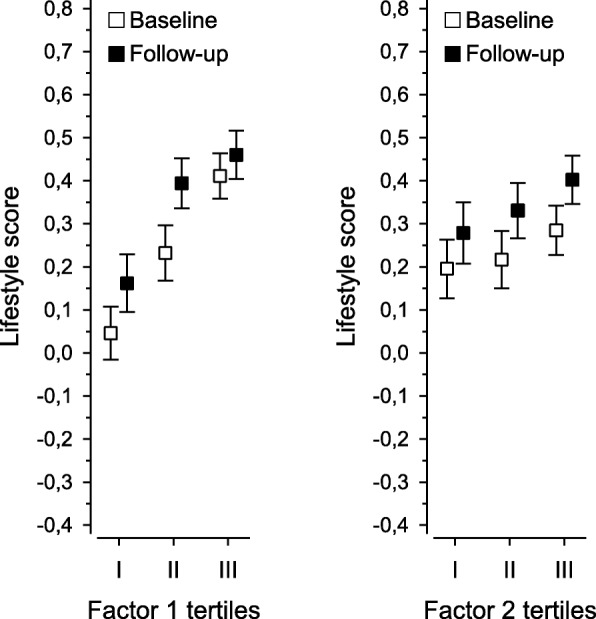
Lifestyle at the baseline and the change after a 5-year follow-up in tertiles of Factor 1 (underrating/resistant) and Factor 2 (helpless/pessimistic)

Figure 1 represents the lifestyle score at the baseline and follow-up and the proportions of those who improved their lifestyle in the follow-up according to tertiles of Factor 1 (underrating/resistant) and Factor 2 (helplessness/pessimism). Indicating a more favorable lifestyle associated with less underrating/resistant attitudes, there was a positive linear trend in the baseline lifestyle score between tertiles of Factor 1 (*P* < 0.001, adjusted with age, sex, education years, BDI-score and BMI). In the follow-up, a positive change of lifestyle score was found in all tertiles of Factor 1 (tertile I, *P* < 0.001; tertile II, *P* < 0.001; tertile III, *P* = 0.035, adjusted with age, sex, education years, BDI-score, BMI and lifestyle index at baseline). There was a significant difference between the proportions of those who could improve their lifestyle (*P* = 0.048). This proportion was the smallest in tertile I representing those with the most underrating/resistant attitudes.

Respectively for Factor 2 (helplessness/pessimism), the baseline lifestyle score was smaller in tertiles I and II compared with tertile III but the difference between tertiles was non-significant (*P* = 0.11, adjusted with age, sex, education years, BDI-score and BMI). However, there was a significant positive change in all tertiles (tertile I, *P* < 0.006; tertile II, *P* < 0.001; tertile III, *P* < 0.001, adjusted with age, sex, education years, BDI-score, BMI and lifestyle index at baseline). The difference between the proportions of those who improved their lifestyle was non-significant (*P* = 0.44).

**Fig. 2:**
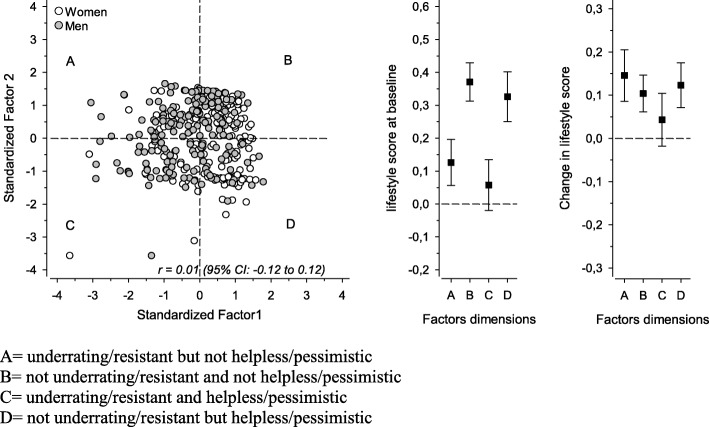
Scores of Factor 1(underrating/resistant) and Factor 2 (helpless/pessimistic) and lifestyle at the baseline and the change after a 5-year follow-up according to attitude dimensions

To further study the attitudes and beliefs, four dimensions based on Factor I and Factor 2 were calculated: A = underrating/resistant but not helpless/pessimistic; B = not underrating/resistant and not helpless/pessimistic; C = underrating/resistant and helpless/pessimistic; D = not underrating/resistant but helpless/pessimistic. There was no correlation between the two factors. Figure 2 shows the distribution of participants according to these dimensions in women and men. The proportion of men and women were different in the four quadrants (*P* < 0.001). Of the women, 34% were in quadrant B and 14% in quadrant A. 32% of the men were in quadrant A and 18% in quadrant D. At the baseline, there was a significant (age and sex standardized, *P* < 0.001) difference in lifestyle between these groups. According to the dimensions, the least healthy lifestyle was found in dimension C. Subjects of dimension B had the healthiest lifestyle. At the follow-up, there was not a significant difference in lifestyle change between these groups (baseline lifestyle, age and sex standardized, *P* = 0.09). However, all groups except C (underrating/resistant and helpless/pessimistic) improved their lifestyles significantly. The underrating/resistant but not helpless/pessimistic (dimension A) subjects had the most significant positive lifestyle change.

## Discussion

This community-based follow-up study indicates that health beliefs and attitudes have a significant association with lifestyle and an impact on lifestyle change. In general, subjects who underrate the risks and have a resistant attitude towards health promotion seemed to have unhealthier lifestyle compared to others. However, subjects with the trait of risk underrating and resistance without pessimistic views and helplessness had the most remarkable potential to improve their lifestyle. Contrary to that, the resistant underraters who were also pessimistic had the unhealthiest lifestyle and did not change their lifestyle at all.

Two different types of attitude characteristic factors explained 89% of the total variance. Factor 1 (underrating/resistant), in which the included items reflect negative or denial attitude to healthy lifestyle or health promotion, and Factor 2 (helplessness/pessimism) in which the items reflect a pessimistic attitude to their ability to influence their health. Resistant and underrating views were associated with higher levels of cardiovascular risk factors and depressive symptoms. Factor 2 (helplessness/pessimism) did not have a similar relationship with cardiovascular risk factors. Lifestyle and lifestyle changes were analyzed taking age, sex, education years, BDI-score and BMI (and in the analysis of change also lifestyle score at baseline) into account as potential confounders. Regardless of these, significant differences regarding lifestyle between the tertiles of Factor 1 and 2 were found. The internal consistency of each factors was moderate, indicating that different items measured the same phenomenon.

Based on the factor analysis, four different attitude dimensions could be constructed. The groups defined by these dimensions were different regarding their lifestyle and lifestyle change. People who do not underrate risks and resist health promotion did not seem to be a big challenge. They had at least a moderately healthy lifestyle at the baseline and they could also improve it. Surprisingly, the resistant underraters without pessimism and helplessness were able to improve their lifestyle significantly. These findings indicate that, in general, there are not grounds for pessimism or nihilism in health promotion itself. It has been stated that knowledge of health risks is the prerequisite for lifestyle change [19]. Most people could have the ability, at least to some extent, to respond to health promotion actions but perhaps not those with resistant and pessimistic attitudes and beliefs.

A recent study indicated that pessimism is independently associated with the risk for coronary heart disease in men [20]. Earlier studies have shown that optimism is associated with a healthier lifestyle and a lower level of cardiovascular risk factors [21, 22]. It was not possible to assess optimism in this study but it can be assumed that people in tertile III of Factor 2 were more optimistic (less pessimistic) than those in tertile I. It has been suggested that to target preventive actions, pessimism should be measured and there may also be ways to do it that are practical and do not take a lot of time [21]. We do not yet know whether Factor 2 in the present study assesses pessimism and helplessness alone. Neither do we know yet what other features in addition to resistant attitudes and underrating of risks are associated with Factor 1. However, the present study indicates that it is possible to profile people with different probabilities for lifestyle change.

The groups of people whose lifestyle is the unhealthiest and who are least capable of change may represent at least to some extend subjects with a low level of perceived self-efficacy. Perceived self-efficacy has been defined as the ability to exercise control over one’s health habits [19]. The findings of the present study suggest that one reason for perceived difficulties in health promotion may be that subjects to whom it should be targeted have the lowest readiness to change. This was also found in a multinational survey that indicated that many primary care patients who have unhealthy lifestyles do not perceive a need for lifestyle change [23]. Based on the present study, we are not able to say whether there are practical possibilities to impact their health behavior or not. However, in order to target health promotion, it could be possible to detect these most challenging subjects and tailor the support and health promotion activities more personally. Instead of traditional health promotion, psychosocial support and actions supporting self-efficacy could be better options for people with the unhealthiest lifestyles and resistant and pessimistic views. Interventions based on e.g. social cognitive theory [19], the transtheoretical model of change [24], or self-affirmation on health-behavior change [25] may be beneficial in understanding resistance towards beneficial health information and in reducing it. The essential goal is to implement functioning theory-based health promotion in everyday primary care actions [11]. However, the current health promotion strategies do not seem to be beneficial in practice. At least people with negative attitudes and especially those feeling helpless or pessimistic need additional strategies. For at least some specific target groups, practices including elements of mindfulness may be useful [26]. Health promotion practices based on a social cognitive approach are promising on a larger scale [19, 27].

The main strength of this study is its wide coverage of a single community. Because of the moderately high rate of participation, the population of the health survey represents a typical semirural community in eastern Finland. However, the number of participants was not very high and they do not represent a larger area. Therefore, the generalization of these results to the whole population is not yet justified before conducting a larger population-based study. The follow-up and a repetitious comprehensive measurement of lifestyle, which enable the measurement of change, can be regarded as a strength of the present study. One limitation of this study was the fact that we were not able to assess a change in attitudes during the follow-up. Although the lifestyle assessment was comprehensive, it was based on self-reporting, which may be prone to reporting bias. The participants did not receive any particular intervention. However, each of the participants received personal feedback and a recommendation based on their health examination results. Rather than to study the effect of an intervention, our aim was to assess the relationship of health behavior change with attitudes and beliefs in a cohort of the general population with a 5-year follow-up. Principally it can be assumed that those who participated in the baseline and follow-up measurements were more interested in their health than those who did not participate. Plausibly the participants received a signal promoting lifestyle change but the difference of the impact was determined by their attitudes and beliefs.

## Conclusions

This study indicates that lifestyle change is possible among the subjects in the community. People underrating risks and resisting health promotion with pessimistic views towards health behavior change have the unhealthiest lifestyles and the poorest capacity to improve it. These findings suggest that it is possible to identify different groups of people with different needs and readiness and ability to change their health behavior.

## Data Availability

The data generated during this study are not public because availability was not included in the study plan approved by the ethics committee and in the informed consent obtained from the participants. However, the data are available from the corresponding author on reasonable request.

## Abbreviations

ANCOVA: Analysis of covariance
ANOVA: Analysis of variance
BDI: 21-item Beck Depression Inventory
BMI: Body mass index
EUROPREV: The European Network on Prevention and Health Promotion
HAPA: Health Action Process Approach
HDL: High density lipoprotein
MONICA: Monitoring trends and determinants in cardiovascular disease
SD: Standard devition
